# Characterization of hyaluronan-coated extracellular vesicles in synovial fluid of patients with osteoarthritis and rheumatoid arthritis

**DOI:** 10.1186/s12891-021-04115-w

**Published:** 2021-03-06

**Authors:** Anne-Mari Mustonen, Janne Capra, Kirsi Rilla, Petri Lehenkari, Sanna Oikari, Tommi Kääriäinen, Antti Joukainen, Heikki Kröger, Tommi Paakkonen, Johanna Matilainen, Petteri Nieminen

**Affiliations:** 1 Faculty of Health Sciences, School of Medicine, Institute of Biomedicine, University of Eastern Finland, P.O. Box 1627, FI-70211 Kuopio, Finland; 2 Faculty of Science and Forestry, Department of Environmental and Biological Sciences, University of Eastern Finland, P.O. Box 111, FI-80101 Joensuu, Finland; 3 Faculty of Health Sciences, School of Medicine, Institute of Biomedicine, Cell and Tissue Imaging Unit, University of Eastern Finland, P.O. Box 1627, FI-70211 Kuopio, Finland; 4 Faculty of Medicine, Cancer and Translational Medicine Research Unit, University of Oulu, P.O. Box 5000, FI-90014 Oulu, Finland; 5 Department of Surgery and Medical Research Center, Oulu University Hospital, P.O. Box 21, FI-90029 Oulu, OYS Finland; 6 Department of Orthopaedics, Traumatology and Hand Surgery, Kuopio University Hospital, P.O. Box 100, FI-70029 Kuopio, KYS Finland

**Keywords:** Extracellular vesicles, Hyaluronan, Osteoarthritis, Rheumatoid arthritis, Synovial fluid

## Abstract

**Background:**

Hyaluronic acid (HA) is the major extracellular matrix glycosaminoglycan with a reduced synovial fluid (SF) concentration in arthropathies. Cell-derived extracellular vesicles (EV) have also been proposed to contribute to pathogenesis in joint diseases. It has recently been shown that human SF contains HA-coated EV (HA–EV), but their concentration and function in joint pathologies remain unknown.

**Methods:**

The aim of the present study was to develop an applicable method based on confocal laser scanning microscopy (CLSM) and image analysis for the quantification of EV, HA-particles, and HA–EV in the SF of the human knee joint. Samples were collected during total knee replacement surgery from patients with end-stage rheumatoid arthritis (RA, *n* = 8) and osteoarthritis (OA, *n =* 8), or during diagnostic/therapeutic arthroscopy unrelated to OA/RA (control, *n* = 7). To characterize and quantify EV, HA-particles, and HA–EV, SF was double-stained with plasma membrane and HA probes and visualized by CLSM. Comparisons between the patient groups were performed with the Kruskal–Wallis analysis of variance.

**Results:**

The size distribution of EV and HA-particles was mostly similar in the study groups. Approximately 66% of EV fluorescence was co-localized with HA verifying that a significant proportion of EV carry HA. The study groups were clearly separated by the discriminant analysis based on the CLSM data. The intensities of EV and HA-particle fluorescences were lower in the RA than in the control and OA groups.

**Conclusions:**

CLSM analysis offers a useful tool to assess HA–EV in SF samples. The altered EV and HA intensities in the RA SF could have possible implications for diagnostics and therapy.

**Supplementary Information:**

The online version contains supplementary material available at 10.1186/s12891-021-04115-w.

## Introduction

Hyaluronic acid (HA) is a high-molecular-weight (HMW) glycosaminoglycan of the extracellular matrix (ECM) with different functions in tissue injury and repair [1]. It is synthesized by plasma membrane-anchored HA-synthases (HAS1–3) that release the newly-synthesized HA into the ECM. HA incorporates to the pericellular HA coat by remaining attached to HAS or by binding to cell surface receptors. In synovial joints, synoviocytes secrete HMW–HA into synovial fluid (SF), where it functions as a lubricating and anti-inflammatory substance [2]. Inflammation and oxidative stress accelerate the degradation of HA by reactive oxygen species and hyaluronidases [2, 3]. Low-molecular-weight HA is abundant at sites of active tissue catabolism and it promotes inflammation [4]. Circulating HA concentrations increase in many disease states and, in the case of joint disorders, they are elevated in patients with rheumatoid arthritis (RA) compared to those with osteoarthritis (OA) and healthy controls [5, 6]. In SF, HA concentrations decrease more clearly in RA than in OA, and the molecular weight distribution is shifted towards lower ranges in both arthropathies [7].

Extracellular vesicles (EV) are nanosized membrane-coated particles that virtually all cell types secrete into biological fluids [8]. They are often classified into exosomes (30–250 nm in diameter), microvesicles (100–1000 nm), and apoptotic bodies (1–5 μm), with different modes of biogenesis. In this report, EV refers to all types of nanosized vesicles present in body fluids. The shedding of EV is a continuous process that is stimulated by inflammation, tissue renewal, and cancer progression [8, 9]. EV represent their cellular origin by transporting membrane and cytosolic molecules as well as HA synthesis machinery as bioactive cargo and they function in intercellular signaling. As EV can carry CD44—a major receptor for HA—on their surface, HA could provide a link between EV and the HA-rich ECM.

EV can be important mediators in inflammatory and autoimmune diseases by displaying pro-inflammatory activities and by contributing to hypercoagulation [10–12]. Several rheumatic diseases are associated with increased EV numbers [11]. Different EV types increase in the plasma of RA patients and they can correlate positively, or sometimes inversely, with the disease activity [13–16]. RA SF also contains higher numbers of EV than OA SF [12, 17, 18]. EV can amplify inflammatory processes in synovial joints by transporting bioactive molecules, such as arachidonic acid, and by stimulating fibroblast-like synoviocytes (FLS) to release cytokines and other mediators of inflammation [8, 19]. They also transport and induce the production of cartilage-degrading proteinases contributing to joint damage. In OA, EV participate in the pathologic mineralization of the articular cartilage.

Both HA and EV are abundant in SF [18, 20], and it has been documented in humans that HA synthesized by FLS can be carried on the surface of EV [21]. Many other cell types, such as mesenchymal stem cells, primary mesothelial cells, and melanoma cells, also release HA-coated EV (HA–EV) [22–24]. They have been proposed to function, for instance, in cell–cell interactions, ECM remodeling, and tissue regeneration. Regarding joint diseases, HA–EV concentrations could both reflect and potentially affect different disease states and, thus, the understanding of the regulation of HA–EV secretion would have potential use in therapeutic applications of orthopedic diseases. By establishing clinically relevant threshold concentrations, HA–EV could be utilized as biomarkers for diagnosis, disease activity, prognosis, and treatment of different arthropathies. Local HA injections are widely used as treatments for OA but their clinical effectiveness remains controversial [25]. The precise mechanisms of action by which HA protects articular cartilage are still unclear, but HA is known to improve the viscoelastic properties of SF [20]. It could also display anti-inflammatory activities [26], affect proteoglycan synthesis and release [27, 28], act as a barrier against catabolic substances on the cartilage surface [28], and suppress the production of cartilage-degrading enzymes via binding to CD44 [29, 30]. Stimulation of endogenous HA production in the form of HA–EV could have benefits over industrially-produced preparations, for instance, by making it possible to avoid contamination by exogenous biological material.

The aim of the present study was to develop an applicable method for the quantification of HA–EV in the SF of the human knee joint for both basic and translational research. It was hypothesized that the quantity of HA–EV would reflect different joint disorders, especially regarding the more inflammatory nature of RA compared to OA [31].

## Methods

### Subjects, ethics, and sampling

Patients with knee joint disorders (seropositive RA: men *n* = 3, women *n* = 5; primary OA: men *n* = 1, women *n* = 7; Table 1) were recruited at the Oulu University Hospital with the permission of the Ethical Committee of the Hospital (decision #29/2011, amendment 2/24/2014) in compliance with the Helsinki Declaration. As the selection criteria, only patients who were ≥ 18 years of age and undergoing knee surgery for pre-existing medical indications were sampled (operative diagnoses listed in Supplementary file 1). Prior to the surgery, the patients signed informed consent forms to donate their SF samples. Demographic data were recorded as follows: sex, age, body mass, height, body mass index (BMI), operation, operative diagnosis, and medication. Previous HA injections that two of the OA patients had received were not considered an exclusion criterion. No data that would enable the identification of the patients were recorded.

**Table 1:** General characteristics of the sampled knee surgery patients (mean ± SE)

Group	Control	RA	OA	*p*
Gender	4 M, 3F	3 M, 5F	1 M, 7F	0.226
Age	33 ± 4^A^	71 ± 3^B^	67 ± 2^B^	0.001
Body weight	79.8 ± 6.7	70.9 ± 6.9	86.4 ± 6.3	0.277
BMI	26.8 ± 2.1	25.3 ± 1.8	32.2 ± 2.2	0.084

*RA* rheumatoid arthritis, *OA* osteoarthritis, *M* male, *F* female, *BMI* body mass index; sex ratios were tested with the Fisher's exact test, means with dissimilar superscript letters indicate significant differences between the study groups within a row, those with similar superscript letters are not significantly different from each other (Kruskal–Wallis ANOVA)

SF samples were collected with sterile needles and syringes from fasted patients during total knee replacement surgery performed at the Oulu University Hospital in 2011–2017 and stored at − 70 °C. All samples were obtained during surgery for pre-existing indications and they represented tissue that would have been removed during surgery regardless of the study. The total duration of the surgery was increased only by a minimal amount of time due to sampling. Control SF samples (men: *n* = 4, women: *n* = 3) were collected during arthroscopic knee surgery performed as diagnostic arthroscopy or due to non-OA/RA-related trauma at the Kuopio University Hospital in 2014–2018 with the permission of the Ethical Committee of the Hospital (decision #79//2013, #73/2016) in compliance with the Helsinki Declaration. The selection criteria for the patients, the collection of general data, and the handling of the SF samples were similar to patients with RA or OA. The required sample size was determined with Mead's resource equation.

### Confocal laser scanning microscopy (CLSM)

To visualize HA on EV, a fluorescent group (Alexa Fluor™ 594, A10239; Thermo Fisher Scientific, Waltham, MA, USA) was coupled to the HA-binding complex (HABC) [32]. CellMask™ Deep Red Plasma membrane Stain (Thermo Fisher Scientific; 1:1000) was soluted together with Alexa Fluor™ 594-labeled HABC stain (10 μg/ml) in PBS to double-label the plasma membranes of EV simultaneously with HA. The SF samples were incubated with the HA and plasma membrane probe solution for approximately 30 min at room temperature and imaged on 8-well ibidi microscopy chambers (ibidi GmbH, Martinsried, Germany). The specificity of the stainings was controlled by samples with only SF and buffer and probes and buffer, respectively. Imaging was performed with the Zeiss Axio Observer inverted microscope equipped with a Zeiss LSM 800 confocal module (Carl Zeiss MicroImaging GmbH, Jena, Germany). Initial image acquisition was carried out using the ZEN 2 (blue edition) software (Carl Zeiss MicroImaging GmbH). A total of 10 images were taken from each sample.

We also used CD63 antibody (353037, BioLegend, San Diego, CA, USA) and phalloidin-iFluor (ab176757, Abcam, Cambridge, UK), conjugated to Alexa Fluor™ 488 and 594, respectively, to validate that the visualized structures were actual EV [33]. CD63 staining was performed on unprocessed SF similar to HA and plasma membrane probes. For phalloidin staining, SF was diluted 1:5 with sterile-filtered PBS (0.22 μm pore size). To remove cell debris, the sample was first centrifuged at 1000 *g* for 10 min at + 4 °C, and the supernatant was transferred into a new tube and centrifuged at 1200 *g* for 20 min at + 4 °C. Finally, the supernatant was ultracentrifuged at < 110,000 *g* for 90 min at + 4 °C, and the obtained EV pellet was suspended in PBS and stored at − 80 °C. The enriched EV were placed on ibidi coverglasses coated with 10 μg/ml Poly-D-lysine hydrobromide (P6407, Sigma-Aldrich, St. Louis, MO, USA) at + 37 °C in 5% CO_2_ overnight. Prior to the addition of phalloidin-iFluor, the EV were treated with 0.1% Triton X-100–1% BSA to increase the permeability of the EV membrane.

The area and intensity of the stainings (CellMask™ Deep Red Plasma membrane Stain, Alexa Fluor™ 594, and combined), count of EV, HA-particles, and HA–EV, and size distribution of EV and HA-particles were determined with ImageJ/Fiji software (NIH, Bethesda, MA) with various open-source plug-ins. From all confocal images, Background Subtraction was performed by using the Rolling Ball radius of 50 pixels and the Sliding Paraboloid setting. Subsequently, Gaussian Blur using Sigma (radius) 1.00 was applied for improved segmentation of the particles. For the particle analysis, the different color channels were first separated and then an automated Threshold was applied to each color channel using the MaxEntropy method. Once Thresholding had been performed, the images were ready for the measurements using the Analyze Particles tool with the size in pixel units set to 5–∞ and the Include Holes command switched on. The areas and intensities of the plasma membrane stain signal were utilized to estimate the lipid component of EV. In further sections of this report, this lipid component of EV is simply referred to as EV. Co-localization analysis was performed using the Coloc2 tool with the Costes Threshold Regression and 100 Costes randomisations. The number of co-localized particles from each image was counted using the ComDet plug-in with default settings. Using the results from the ComDet analysis, each EV-particle that co-localized with HA was identified, and the diameters (nm) of these particles were measured.

### HA concentration and molecular weight distribution in SF

Two control patients could not be studied for SF HA concentrations as the whole sample volume left was utilized for the CLSM analyses with higher priority. For HA determinations (*n* = 5, 8, and 8 for control, RA, and OA, respectively), 5 μl of SF was diluted 1:100 with 150 mM sodium acetate (pH 6.8) and treated with proteinase K (300 μg/ml, Thermo Fisher Scientific) at + 50 °C overnight. Thereafter the samples were heated to + 98 °C for 20 min and centrifuged at 16,000 *g* for 15 min at + 4 °C. The supernatant was collected and used either for total HA analysis with a sandwich-type enzyme-linked sorbent assay (ELSA) [34] or for HA molecular weight distribution determination [35]. For total HA analysis, the samples were further diluted with 1% BSA–PBS, final dilution varied from 1:40,000 to 1:60,000. HA size was determined with size-exclusion chromatography using a Sephacryl S-1000 (1 × 30 cm) column and 100 mM NH_4_HCO_3_ as the running buffer. The column was calibrated with 2500 kDa HA, 150 kDa HA (Hyalose, Oklahoma City, OK, USA), and glucuronic acid (Sigma-Aldrich). The proteinase K-treated samples were further diluted with PBS (1:2500) and injected into a column. From each sample, 25 fractions with a volume of 1 ml were collected and lyophilized. The dried fractions were dissolved into 1% BSA–PBS and analyzed for their HA content by ELSA.

### Statistical analyses

Comparisons of the variables between the patient groups were performed with the Kruskal–Wallis analysis of variance (ANOVA; IBM SPSS *v*25 software, IBM, Armonk, NY, USA). Differences between genders were tested with the Mann–Whitney U test. Nonparametric tests were selected due to the relatively small sample size. Sex ratios in the study groups were compared with the Fisherʼs exact test, and correlations were calculated with the Spearman correlation coefficient (r_s_). The *p* value < 0.05 was considered statistically significant. The results are presented as the mean ± SE. To perform a general assessment of the CLSM results, we also conducted the linear discriminant function analysis (IBM SPSS *v*25 software), which allows the simultaneous analysis of several variables and classifies the differences between two or more groups of objects. It estimates the relationship between a single grouping variable (diagnosis in this case) and a set of independent discriminating variables (CLSM data). The analysis revealed, how the samples in the three diagnosis groups differed from one another, which variables separated the diagnoses most clearly, and how well the analysis was able to classify the samples into their respective diagnoses.

## Results

The average ages were higher for the RA and OA groups compared to the controls (Table 1). The sex ratios, body masses, and BMI did not differ between the study groups. There were no differences in the SF HA concentrations (control: 1.7 ± 0.43; RA: 1.2 ± 0.19; OA: 1.8 ± 0.24 mg/ml; Kruskal–Wallis ANOVA, *p* = 0.149), nor in its molecular weight distribution between the diagnoses (Kruskal–Wallis ANOVA, *p* = 0.566–0.786; Supplementary file 2).

**Fig. 1:**
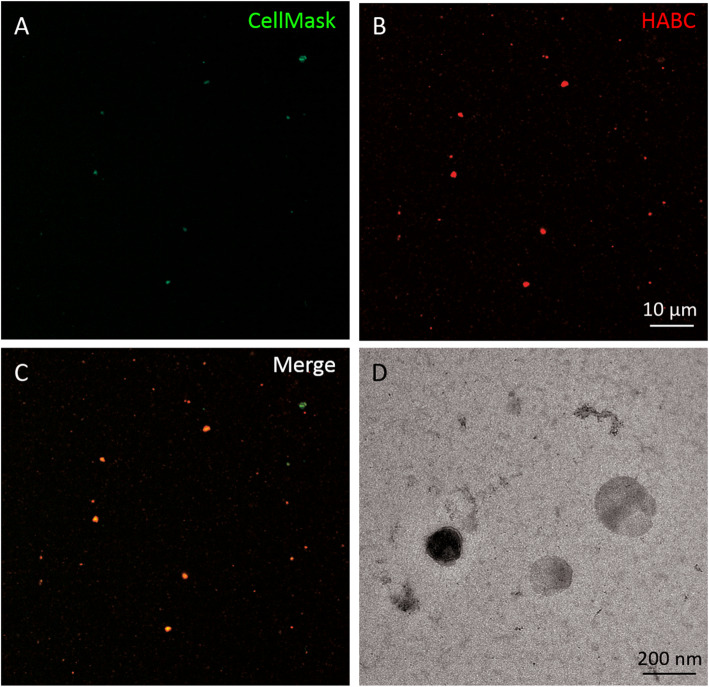
Synovial fluid stained with CellMask™ Deep Red Plasma membrane Stain and fluorescent hyaluronan (HA) binding complex (HABC). An unprocessed synovial fluid sample stained with CellMask™ Deep Red Plasma membrane Stain (pseudocolored green) in (panel **a**), Alexa Fluor™ 594-labeled fluorescent HABC (pseudocolored red) in (panel **b**), and a merged image in (panel **c**). HA-containing particles not associated with vesicle membranes are also visible. Panel **d** represents electron microscopic ultrastructure of vesicles in synovial fluid. The processing and visualization of the sample for electron microscopy were performed as outlined previously [21]

**Fig. 2:**
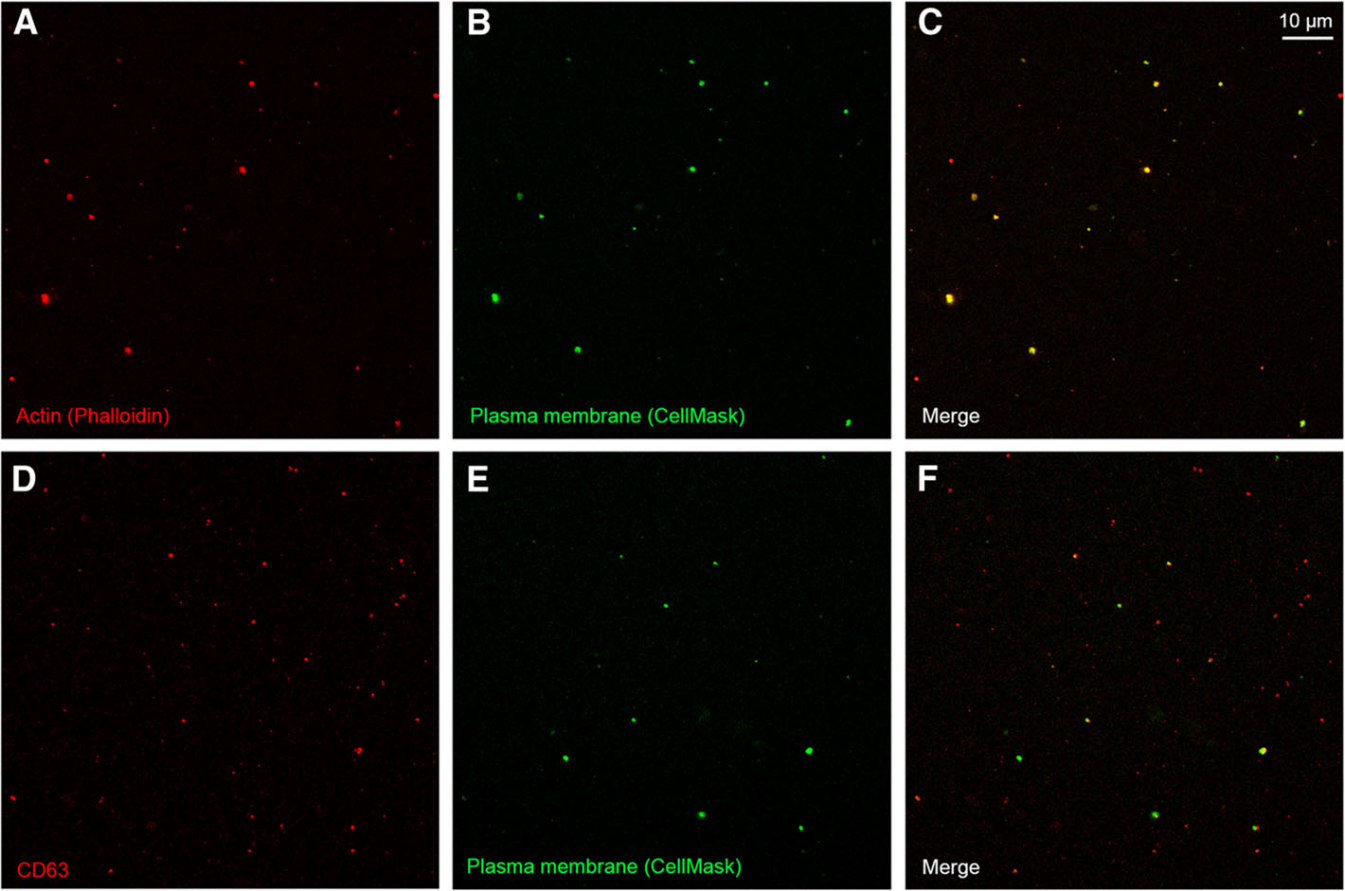
Synovial fluid stained with fluorescent phalloidin, CD63, or CellMask™ Deep Red Plasma membrane Stain. An ultracentrifuged synovial fluid sample stained with Alexa Fluor™ 594-labeled phalloidin (pseudocolored red) and CellMask™ Deep Red Plasma membrane Stain (pseudocolored green) in (panels **a** and **b**), an unprocessed synovial fluid sample stained with Alexa Fluor™ 488-labeled fluorescent CD63 (pseudocolored red) or CellMask™ Deep Red Plasma membrane Stain (pseudocolored green) in (panels **d** and **e**), and merged images in (panels **c** and **f**) depicting the co-localization of the stains

**Fig. 3:**
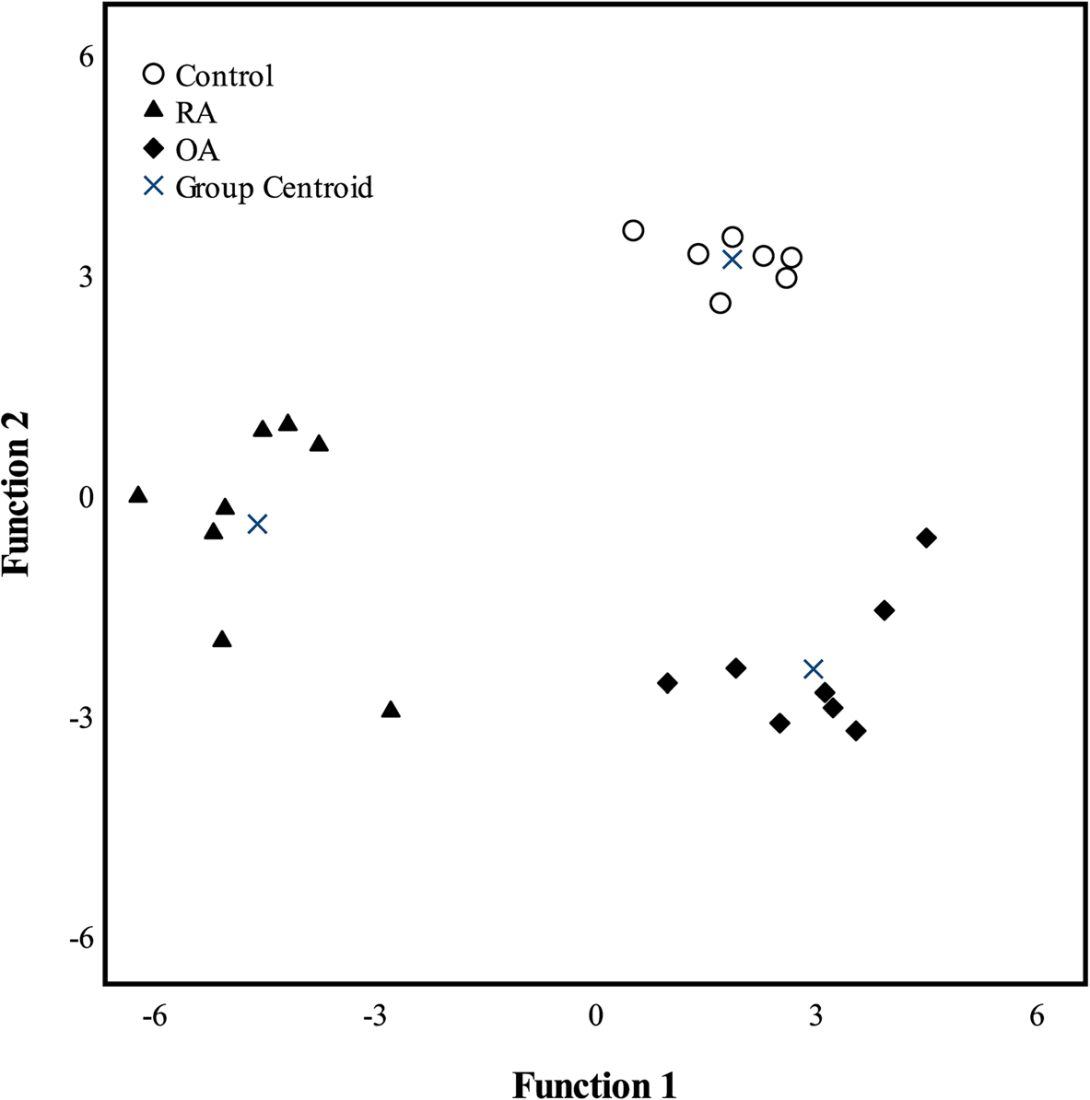
Discriminant analysis of confocal microscopy data from synovial fluid of the patient groups. Discriminant analysis depicting the classification of confocal microscopy data from synovial fluid of patients with traumatized knees (Control), rheumatoid arthritis (RA), and osteoarthritis (OA) based on discriminant functions 1 and 2. The first function displayed in the x-axis explained 69% of the variance in the dataset and the second function in the y-axis accounted for 31% of the variance. Overall, the analysis classified 100% of samples correctly into their respective diagnoses

CLSM demonstrated that all studied SF samples contained varying numbers of EV, HA-particles, and HA–EV (Fig. 1). Studied particles also contained CD63 and phalloidin co-localized with the plasma membrane stain (Fig. 2). In the discriminant analysis, the three study groups were clearly separated from each other based on the CLSM data (Fig. 3). The most interesting variables separating the groups included the intensity of EV (i.e., membrane-fluorescent material), intensity of HA-particle fluorescence, area of HA-particles, count of HA–EV, and count of EV. The first function in the x-axis explained 69% of the variance in the dataset and the second function in the y-axis accounted for 31% of the variance. The analysis classified 100% of the samples correctly into their respective study groups.

**Fig. 4:**
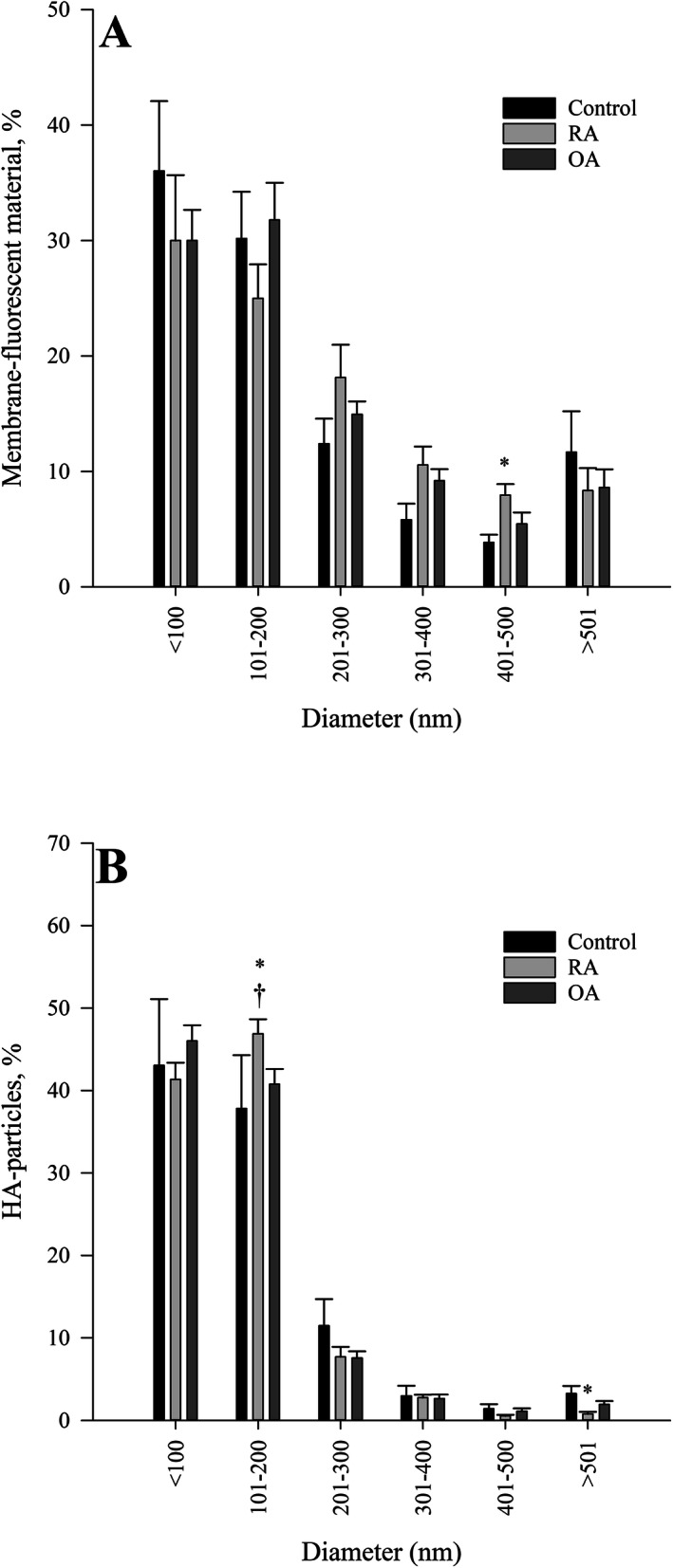
Size distribution of extracellular vesicles and hyaluronan (HA)-positive particles in human synovial fluid. Size distribution of membrane-fluorescent material (i.e., extracellular vesicles) in (panel **a**) and HA-positive particles in (panel **b**) in unprocessed human synovial fluid samples analyzed with confocal laser scanning microscopy. RA = rheumatoid arthritis, OA = osteoarthritis, * significant difference from control, † significant difference from OA (Kruskal–Wallis ANOVA, *p* < 0.05)

The size distribution of EV and HA-particles was for the most part similar in the study groups (Fig. 4a–b). The average percentages of EV of different sizes were 31.8% for ≤100 nm, 28.9% for 101–200 nm, 15.3% for 201–300 nm, 8.6% for 301–400 nm, 5.8% for 401–500 nm, and 9.5% for ≥501 nm in diameter. The RA group had a higher proportion of EV of 401–500 nm in diameter than the control group. The corresponding values for HA-particles were as follows: 43.5% (≤100 nm), 42.0% (101–200 nm), 8.8% (201–300 nm), 2.8% (301–400 nm), 1.0% (401–500 nm), and 1.9% (≥501 nm in diameter). The RA group had a higher proportion of HA-particles of 101–200 nm in diameter than the other groups but a lower proportion of particles of ≥501 nm in diameter than the control group. Approximately 66% of EV fluorescence was co-localized with HA (Table 2; Fig. 1). The count and diameter of HA–EV averaged 48 ± 5 and 367 ± 16 nm, respectively, with no differences between the groups. The intensities of EV and HA-particles were lower in the RA than in the control and OA groups.

**Table 2:** Confocal microscopy data of the sampled knee surgery patients (mean ± SE)

	Control (*n* = 7)	RA (*n =* 8)	OA (*n* = 8)	*p*
Area of EV, μm^2^/visual field	63.4 ± 14.6	48.8 ± 6.5	56.4 ± 6.5	0.726
Intensity of EV, AU	49.1 ± 5.5^B^	29.8 ± 2.0^A^	43.4 ± 2.7^B^	0.003
Count of EV, N	101 ± 11	82 ± 14	93 ± 10	0.463
Area of HA-particles, μm^2^/visual field	20.2 ± 4.1	12.9 ± 0.7	18.0 ± 2.1	0.272
Intensity of HA-particles, AU	41.4 ± 5.9^B^	25.7 ± 1.7^A^	41.7 ± 2.6^B^	0.002
Count of HA-particles, N	231 ± 47	212 ± 22	204 ± 18	0.763
Count of co-localized EV and HA-particles, N	46 ± 14	39 ± 5	58 ± 7	0.224
Diameter of co-localized EV and HA-particles, nm	401 ± 36	379 ± 25	324 ± 14	0.110
Co-localization of EV and HA-particles, %	67.0 ± 2.5	66.5 ± 1.6	65.8 ± 1.3	0.854

*RA* rheumatoid arthritis, *OA* osteoarthritis, *EV* extracellular vesicle, *AU* arbitrary unit, *HA* hyaluronan; means with dissimilar superscript letters indicate significant differences between the study groups within a row, those with similar superscript letters are not significantly different from each other (Kruskal–Wallis ANOVA)

As two OA patients had received HA injections, the Kruskal–Wallis ANOVA and discriminant analysis were also performed by excluding these individuals, and it was observed that the exclusion did not change the results of the OA group. All variables, in which there were statistically significant differences according to diagnosis, remained significant after the exclusion of these patients. In the discriminant analysis, the three study groups were clearly separated from each other. The most important variables separating the groups remained the same despite the exclusion of the two cases; functions 1–2 explained 100% of the variance in the dataset, and the analysis classified 100% of the samples correctly into their respective diagnoses.

When the study groups were pooled together, the area of EV fluorescence correlated positively with the area of HA fluorescence (r_s_ = 0.701, *p* < 0.0004) and diameter of HA–EV (r_s_ = 0.580, *p* = 0.004). The EV count correlated positively with the HA-particle count (r_s_ = 0.611, *p* = 0.002) and HA–EV count (r_s_ = 0.600, *p* = 0.002). The count of HA-particles also correlated positively with the HA–EV count (r_s_ = 0.735, *p* < 0.0004). The intensity of EV fluorescence correlated positively with the intensity of HA fluorescence (r_s_ = 0.758, *p* < 0.0004), area of HA-particles (r_s_ = 0.444, *p* = 0.034), and HA–EV count (r_s_ = 0.493, *p* = 0.017). The intensity of HA fluorescence also correlated positively with the HA–EV count (r_s_ = 0.557, *p* = 0.006). The age of the patients correlated inversely with the intensity of EV fluorescence (r_s_ = − 0.429, *p* = 0.041), and the intensity of HA fluorescence correlated positively with the BMI (r_s_ = 0.467, *p* = 0.025). There were no statistically significant sex differences in any of the measured variables in the pooled study material.

## Discussion

The present study investigated EV, HA-particles, and HA–EV in the SF of patients with RA and OA by using CLSM and image analysis. The main findings were that *i*) CLSM and image processing offer a useful tool to assess HA–EV in human SF, *ii*) approximately 66% of EV fluorescence was co-localized with HA, *iii*) EV and HA-particle intensities were lower in the RA than in the control and OA groups, and *iv*) the RA group had a higher proportion of smaller HA-particles but a lower proportion of the particles in the largest size category.

Fluorescent stainings with CD63 (membrane protein) and phalloidin that binds to filamentous actin (cytosolic protein recovered in EV) verified that the studied particles were EV [33]. Their size distribution was for the most part similar between the diagnoses. The main populations of EV in the unprocessed SF samples were ≤ 200 nm in diameter by CLSM and, thus, mostly represented exosomes and microvesicles [8]. The observed size distribution was relatively similar to previous results on the SF of human knee after differential centrifugation, in which the main EV populations were ≤ 300 nm in diameter by nanoparticle tracking analysis and ≤ 400 nm in diameter by transmission electron microscopy [21]. However, vesicles up to 2000 nm were previously documented in an unprocessed SF sample by CLSM and differential interference contrast microscopy. The present results also confirmed the earlier size distribution data (80–400 nm) reported for the SF of OA, RA, and juvenile idiopathic arthritis patients [18, 36, 37].

The EV intensities were the lowest in the RA group, while the EV counts did not vary according to diagnosis. These findings could suggest that the vesicle size decreases in RA, but this was not supported by the EV size distribution data. The unchanged EV count differs from earlier literature documenting increased EV numbers in RA [12–16, 18]. EV have been proposed to influence various processes in RA pathogenesis, for instance, to transport pro-inflammatory factors and to promote their secretion by target cells, such as FLS [19]. EV could also induce the release of proteolytic enzymes leading to the degradation of cartilage ECM. As inflammatory processes play a central role in RA [38] and active EV shedding is observed in conditions such as inflammation [9], we expected but were unable to detect increased EV counts in the RA patients. Standardized procedures are still not available for the characterization of EV populations in body fluids due to their small size and heterogeneous nature [39–41]. Because of the diverse methods used for the isolation and detection of EV, the average EV counts are not directly comparable between studies. Many of the earlier investigations utilized flow cytometry with limitations in resolving structures < 200 nm [39]. Moreover, previous studies were mostly conducted on plasma [13–16], where the cellular origin of EV is different from that in SF [10]. SF EV have been studied less intensively in arthropathies, but it has been reported that the levels of EV can be higher in the SF of RA patients compared to those with OA [12, 17, 18]. The unchanged EV counts in the SF of OA patients of the present study confirmed earlier literature with similar EV concentrations in patients with and without OA [42]. In a previous study on the human knee, the SF EV counts were also similar between patients with primary and post-traumatic OA when measured with nanoparticle tracking analysis [21].

The intensity of HA fluorescence was the lowest in the RA group. This finding agrees with previous literature, according to which SF HA levels reduce more pronouncedly in RA and to a lesser degree in OA compared to controls [7]. The lowered intensity of HA fluorescence with a stable particle count could mean an altered distribution of HA into smaller aggregates, which is supported by the size distribution data. The higher proportion of HA-particles of 101–200 nm in diameter and the lower proportion of particles of ≥501 nm in diameter in the RA group are in concordance with the expectation that the molecular weight of HA would reduce in RA [7]. The lowered levels of HMW–HA could be caused by the reduced expression of HAS1–2 and the increased expression of hyaluronidase-2 in RA synovium [43]. Moreover, the increased expression of HAS3, which produces smaller HA polymers than HAS1–2 as well as drives inflammation [44, 45], may also lead to a reduced molecular weight distribution of HA [43]. These changes could impair the viscoelastic properties of SF [20], promote pro-inflammatory activities [4], and increase the production of cartilage-degrading enzymes [29], thus, worsening the pathophysiological processes in the knee joint.

While the visualized HA exhibited the above-mentioned phenomena, the same could not be observed in the biochemical analyses. It is plausible that the different methods used measure somewhat different aspects of HA distribution and concentration. The biochemical analysis of HA fractions includes the removal and purification of HA from the natural SF while CLSM analysis utilized here visualizes the HA-particles in a more in situ manner. It is feasible to hypothesize that the HA visible with CLSM represents aggregations of HA-particles that probably contain several HA molecules of diverse size. From the viewpoint of joint health, the more in situ HA is examined, the more the method presumably represents the real-life situation reflected in the symptomology of the patients. In addition, the CLSM results showed more conformity to previous literature. Thus, we suggest that it offers a novel method to assess the HA status of the joint close to the actual situation in the diseased knee and provides a promising method to correlate HA-particle abundance and size to disease progression.

Approximately 66% of EV fluorescence was co-localized with HA verifying that HA is partly but not exclusively transported on EV [21]. Long HAS-positive protrusions of FLS have been shown to release HA–EV into SF, while chondrocytes and immune cells are other potential cells of origin for their secretion. Hypothetically, HA-coating could also have been formed via HAS [22] or CD44 [24] after EV release into SF, or HA could be contained within EV by the envelopment of HA [46]. The determination of HA–EV is of interest, as currently their functions in synovial joints remain unknown. HA–EV could have future potential as a novel biomarker to assess and predict disease progression in joint disorders. Moreover, if HA–EV are established to have beneficial effects on joint health, it could be possible to utilize artificially formed HA–EV as therapeutic vehicles to distribute HA in inflamed joints, or to promote the endogenous secretion of these particles. To the best of our knowledge, this is the first time HA–EV numbers have been quantified and compared between different joint diseases. Contrary to our hypothesis, the HA–EV counts did not differ between the studied diagnoses and, thus, the possible link between their release and joint diseases will have to be established in the future. One potential explanation for this could be the relatively small sample size. Based on the percentual distribution of EV in different size categories (Fig. 4a), it is clear that their majority was smaller than the average diameter of HA–EV. This indicates that HA was mostly associated with the larger EV. Substantial numbers of HA-particles not associated with vesicle membranes were also detected in the same SF samples. These presumably included free HA and HA-containing protein complexes [47].

As discussed previously, different protocols for the isolation and detection of EV could be one reason for the partly contradictory EV count results. SF is known to be a challenging biological fluid for the isolation of EV by differential ultracentrifugation [48]. We used a novel approach from previous studies and analyzed unprocessed SF from control, RA, and OA patients undergoing knee surgery. HA–EV-particles could be easily detected from small volumes of double-stained SF samples with CLSM. The avoidance of differential centrifugation allowed us to detect the whole population of vesicles. Protein aggregates and immune complexes were not visualized with the stainings used and, thus, they were not confounding factors for the present analysis [36]. In the future, CLSM could offer a quick estimate of the patients' HA reserves in SF to assess if HA supplementation could be useful to treat a particular OA or RA patient. Some potential limitations regarding the present study should be remarked. The relatively small sample size could have resulted in false negatives. The control group did not consist of healthy individuals but patients with traumatized knees unrelated to RA/OA. The control patients were also significantly younger than the RA and OA groups, which was unfortunate, but quite unavoidable considering the demographics of knee trauma vs. degenerative joint diseases. In addition, the RA and OA groups included a relatively small number of men. The potential effects of gender, age, and joint trauma on the levels of EV and/or HA cannot be ignored [49, 50]. Even though the results of the two patients that had received HA injections did not differ from the rest of the OA patients, the injections may have influenced the intraarticular environment. Other confounding factors may include the immunosuppressive therapy used by RA patients that could have influenced their EV levels [16].

In conclusion, the confocal microscopy data clearly separated the control, RA, and OA groups from each other in the discriminant analysis. SF proved to be an important source of information regarding the presence of EV and their association to HA-particles. The most significant finding related to the diagnoses was that RA SF had lower EV and HA intensities than control and OA SF with possible implications for diagnostics and therapy. CLSM and image analysis offer an applicable tool to assess HA–EV in human SF.

## Supplementary Information

**Additional file 1: Supplementary file 1.** Diagnostic data of the sampled knee surgery patients. **Supplementary file 2.** Molecular weight distribution of hyaluronan (HA) in synovial fluid of patients with traumatized knees (Control), rheumatoid arthritis (RA), and osteoarthritis (OA), mean + SE. HMW = high-molecular-weight (≈2500 kDa), MMW = medium-molecular-weight (≈500 kDa), LMW = low-molecular-weight (< 500 kDa). There were no significant differences between the diagnoses (Kruskal–Wallis ANOVA, *p* > 0.05)

## Data Availability

All relevant data analyzed during this study are included in this published article and its supplementary information files.

## Abbreviations

ANOVA: Analysis of variance
BMI: Body mass index
CD44: Cluster of differentiation 44
CLSM: Confocal laser scanning microscopy
ECM: Extracellular matrix
ELSA: Sandwich-type enzyme-linked sorbent assay
EV: Extracellular vesicle
FLS: Fibroblast-like synoviocyte
HA: Hyaluronan
HABC: Hyaluronan binding complex
HA–EV: Hyaluronan-coated extracellular vesicle
HAS: Hyaluronan synthase
HMW: High-molecular-weight
OA: Osteoarthritis
RA: Rheumatoid arthritis
r_s_: Spearman correlation coefficient
SF: Synovial fluid
