# Supplementary material for: Characterization of hyaluronan-coated extracellular vesicles in synovial fluid of patients with osteoarthritis and rheumatoid arthritis
Source: BMC Musculoskelet Disord. 2021 Mar 6;22:247. doi: 10.1186/s12891-021-04115-w (PMC7937210; doi:10.1186/s12891-021-04115-w)
Supplement: Supplementary file 1 — Additional file 1: Supplementary file 1. Diagnostic data of the sampled knee surgery patients. Supplementary file 2. Molecular weight distribution of hyaluronan (HA) in synovial fluid of patients with traumatized knees (Control), rheumatoid arthritis (RA), and osteoarthritis (OA), mean + SE. HMW = high-molecular-weight (≈2500 kDa), MMW = medium-molecular-weight (≈500 kDa), LMW = low-molecular-weight (< 500 kDa). There were no significant differences between the diagnoses (Kruskal–Wallis ANOVA, p > 0.05) [file 12891_2021_4115_MOESM1_ESM.docx]

**Supplementary file 1**

Diagnostic data of the sampled knee surgery patients

| ID | Group | Gender | Operation | Operative diagnosis |
| --- | --- | --- | --- | --- |
| 01 | Control | 1 | Arthroscopy, MPFL reconstruction | M22.0 |
| 02 | Control | 2 | Arthroscopy, ACL reconstruction | M23.5, M23.2 |
| 03 | Control | 2 | Diagnostic arthroscopy | M23.5 |
| 04 | Control | 2 | Arthroscopy, debridement | M25.5 |
| 05 | Control | 1 | Arthroscopy | M22.4 |
| 06 | Control | 1 | Arthroscopy, partial meniscal resection | M23.2 |
| 07 | Control | 1 | Arthroscopy, ACL reconstruction | S83.5 |
| 08 | RA | 2 | Total knee replacement | M17.5 (secondary) |
| 09 | RA | 2 | Total knee replacement | M17.5 (secondary) |
| 10 | RA | 2 | Total knee replacement | M17.5 (secondary) |
| 11 | RA | 1 | Total knee replacement | M17.5 (secondary) |
| 12 | RA | 2 | Total knee replacement | M17.4 (other secondary) |
| 13 | RA | 1 | Total knee replacement | M17.5 (secondary) |
| 14 | RA | 1 | Total knee replacement | M17.5 (secondary) |
| 15 | RA | 2 | Total knee replacement | M17.4 (other secondary) |
| 16 | OA | 1 | Total knee replacement | M17.1 (primary) |
| 17 | OA | 2 | Total knee replacement | M17.1 (primary) |
| 18 | OA | 2 | Total knee replacement | M17.1 (primary) |
| 19 | OA | 2 | Total knee replacement | M17.1 (primary) |
| 20 | OA | 2 | Total knee replacement | M17.1 (primary) |
| 21 | OA | 2 | Total knee replacement | M17.1 (primary) |
| 22 | OA | 2 | Total knee replacement | M17.1 (primary) |
| 23 | OA | 2 | Total knee replacement | M17.1 (primary) |

1 = male, 2 = female, RA = rheumatoid arthritis, OA = osteoarthritis, MPFL = medial patellofemoral ligament, ACL = anterior cruciate ligament

**Supplementary file 2** Molecular weight distribution of hyaluronan (HA) in synovial fluid of patients with traumatized knees (Control), rheumatoid arthritis (RA), and osteoarthritis (OA), mean + SE. HMW = high-molecular-weight (≈2500 kDa), MMW = medium-molecular-weight (≈500 kDa), LMW = low-molecular-weight (<500 kDa). There were no significant differences between the diagnoses (Kruskal–Wallis ANOVA, *p* > 0.05)

**
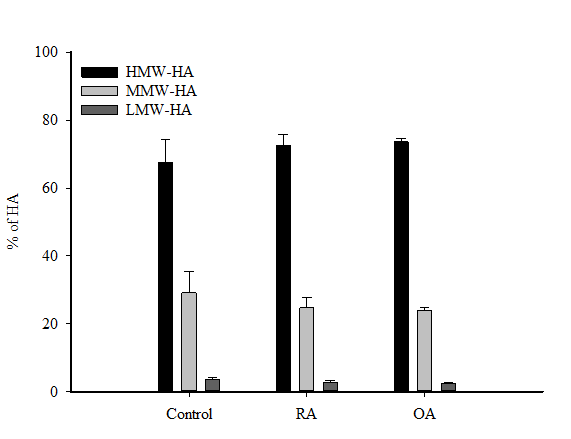
**
